# Evidence for a preformed Cooper pair model in the pseudogap spectra of a Ca_10_(Pt_4_As_8_)(Fe_2_As_2_)_5_ single crystal with a nodal superconducting gap

**DOI:** 10.1038/s41598-019-40528-3

**Published:** 2019-03-08

**Authors:** Y. I. Seo, W. J. Choi, Shin-ichi Kimura, Yong Seung Kwon

**Affiliations:** 1 Department of Emerging Materials Science, DGIST, Daegu, 711-873 Republic of Korea; 2 Graduate School of Frontier Biosciences and Department of Physics, Graduate School of Science, Osaka University, Suita, 565-0871 Japan

## Abstract

For high-*T*_*c*_ superconductors, clarifying the role and origin of the pseudogap is essential for understanding the pairing mechanism. Among the various models describing the pseudogap, the preformed Cooper pair model is a potential candidate. Therefore, we present experimental evidence for the preformed Cooper pair model by studying the pseudogap spectrum observed in the optical conductivity of a Ca_10_(Pt_4_As_8_)(Fe_2_As_2_)_5_ (*T*_*c*_ = 34.6 K) single crystal. We observed a clear pseudogap structure in the optical conductivity and observed its temperature dependence. In the superconducting (SC) state, one SC gap with a gap size of Δ = 26 cm^−1^, a scattering rate of 1/τ = 360 cm^−1^ and a low-frequency extra Drude component were observed. Spectral weight analysis revealed that the SC gap and pseudogap are formed from the same Drude band. This means that the pseudogap is a gap structure observed as a result of a continuous temperature evolution of the SC gap observed below *T*_*c*_. This provides clear experimental evidence for the preformed Cooper pair model.

## Introduction

The BCS theory^1^ announced in 1957 seemed to have revealed a universal law for material superconductivity by enabling understanding of the pairing mechanism for conventional superconductors. However, since it failed to explain the high-*T*_*c*_ cuprate superconductors^2^ discovered in 1986, we have encountered limitations in understanding the universal superconducting mechanism. Additionally, the phenomenon of a Mott insulator^3,4^ or pseudogap^5–7^ observed by the strong correlation in the normal state of the cuprate suggests that the pairing mechanism is caused by a more complicated process than the BCS theory^1^. Much effort has been made to understand this for cuprate superconductors. In particular, many attempts have been made to solve the mystery of the role and origin of the pseudogap seen in the temperature region higher than *T*_*c*_. Pseudogap phenomena have also been found in heavy Fermion superconductors^8^ and more recently in iron-based superconductors^9,10^. This implies that the pseudogap phenomenon is a common phenomenon in unconventional superconductors. Therefore, the correlation of the pseudogap in unconventional superconductors is expected to be closely related to the formation of superconducting electron pairs; the pseudogap mechanism must provide an important clue for understanding the universal pairing mechanism of unconventional superconductors.

There are many theoretical models^11–17^ for explaining the origin of the pseudogap. Since the pseudogap is a partial gap at the Fermi surface that is observed above *T*_*c*_ with an energy scale similar to the energy scale of the superconducting gap, among such models, the preformed Cooper pair model^18,19^ is considered as a potential candidate. The preformed Cooper pair model describes a scenario where Cooper pairs are already present at the superconducting temperature *T*_*c*_ or higher, but superconductivity does not appear due to its fluctuation. Therefore, many studies have been conducted to explain the relationship between superconductivity and the pseudogap phenomenon^5–7^. Recent studies have attempted to understand the pairing mechanism by explaining the pseudogap phenomenon experimentally observed in some iron-based superconductors by applying the theoretical preformed Cooper pair model^9,10^; however, such studies still lack clear experimental evidence to support the preformed Cooper pair scenario. Therefore, we have attempted to explain the pseudogap in the preformed Cooper pair scenario through experimental data obtained by using IR spectroscopy to observe and analyse the pseudogap spectrum.

In this study, we calculated the optical conductivity data by measuring the reflectivity of Ca_10_(Pt_4_As_8_)(Fe_2_As_2_)_5_ (*T*_*c*_ = 34.6 K) single crystals at various temperatures and analysed the pseudogaps observed at temperatures of *T* = 38, 70 and 100 K. We confirmed the temperature dependence of the Drude component and the definite gap spectrum in the low-frequency optical conductivity data for the temperature region where the pseudogap was observed. Through spectral weight analysis and analysis of gap spectrum for the superconducting and pseudogap regions, we demonstrate that the pseudogap is formed as a result of a continuous temperature evolution of the superconducting gap observed below *T*_*c*_. As a result, we provide convincing experimental evidence that the pseudogap in the iron-based superconductor is a phenomenon that can be described by the preformed Cooper pair model.

## Results and Discussions

**Figure 1:**
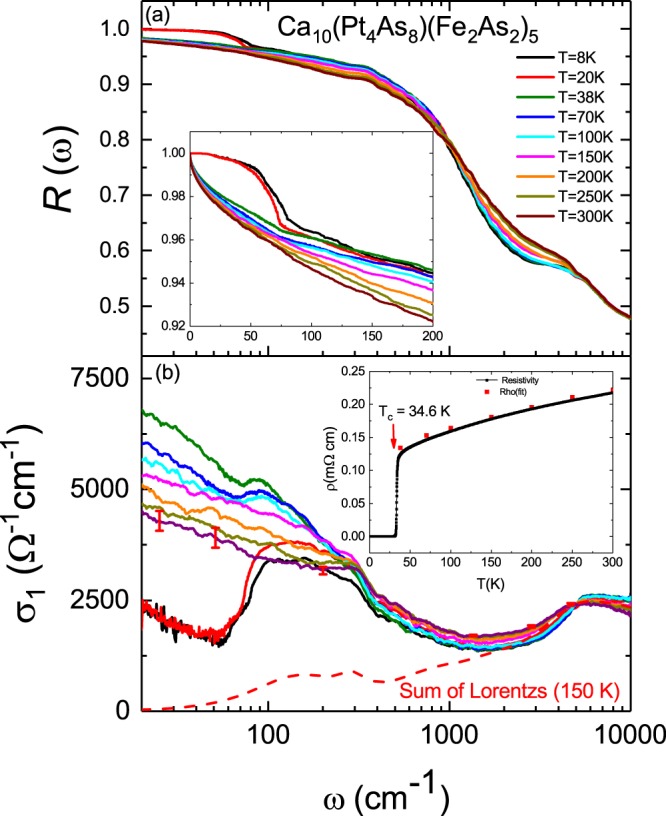
(**a**) Reflectivity spectra *R*(*ω*) of Ca_10_(Pt_4_As_8_)(Fe_2_As_2_)_5_ single crystals at several temperatures. The inset shows an enlarged view of the region below a frequency of 200 cm^−1^. (**b**) Frequency dependence of the real part of the optical conductivity *σ*_1_(*ω*) for a Ca_10_(Pt_4_As_8_)(Fe_2_As_2_)_5_ single crystal at several temperatures. The red dotted line represents the sum of the Lorentz oscillators at *T* = 150 K. The inset represents the temperature dependence of the measured DC electrical resistivity (the black dotted line) data and the DC electrical resistivity (the red dots) calculated from extrapolation of optical conductivity.

Figure 1(a) shows the reflectivity spectrum data measured for Ca_10_(Pt_4_As_8_)(Fe_2_As_2_)_5_ (so-called Ca1048) single crystals from 8 to 300 K. The main panel is shown on a logarithmic scale from 10 to 10000 cm^−1^, and the inset is shown on a linear scale from 0 to 200 cm^−1^. Here, the data from 0 to 20 cm^−1^ are not the measured values but are obtained by extrapolation, as described later. In the normal state (*T* > 38 K), the reflectivity approaches unity as the frequency tends towards zero. This feature is more pronounced as the temperature is lowered, indicating that this compound shows the properties of a typical metal. When the superconducting state is entered at a temperature of 8 and 20 K, the reflectivity of the low-frequency region increases rapidly to a value of unity. However, flat reflectivity patterns such as those in Ba122^9^ and Ca1038^10,20^ iron-based superconductors are not observed. In dirty limit superconductor, it has a flat reflectivity characteristic in the low-frequency region when an *s*-wave type superconducting gap is formed. However, for the case of the Ca1048 compound, flatness in the reflectivity does not appear, indicating that the optical properties of the Ba122 and Ca1038 compounds are unlike those of the Ca1048 compound.

For a more in-depth analysis, we have transformed the reflectivity *R*(*ω*) data into real-valued optical conductivity *σ*_1_(*ω*) data using the Kramers-Kronig (KK) transformation. For the KK transformation, the Hagen-Rubens formula is used in the low-frequency region of the normal state, and (1-*Aω*^4^) is used in the superconducting state. On the other hand, in the region of high frequency above 12000 cm^−1^, the reflectance is constant up to 40 eV and then extrapolated to the free-electron approximation *R*(*ω*) ∝ *ω*^−4^.

Figure 1(b) shows the optical conductivity obtained from the reflectivity on a logarithmic scale in the range of 10 to 10000 cm^−1^. In the low-frequency region of the normal state, the optical conductivity data tend to become narrower as the temperature is lowered from higher temperatures. This is due to the development of a Drude component in the low-frequency range, which is consistent with the metallic properties mentioned previously in the reflectivity data. Near 1000 cm^−1^, the interband transition spectrum begins and reaches up to 10000 cm^−1^. This interband transition spectrum shows a temperature dependence, as also observed in the optical conductivity data obtained for the La-doped Ca1038 sample reported previously^10^. The low-frequency optical conductivities at 38, 70 and 100 K show a hump at 0–100 cm^−1^ along with the Drude spectrum. The simultaneous observation of a hump along with the Drude spectrum above *T*_*c*_ is due to the partial gap formed in the Fermi surface. This is called the pseudogap; a pseudogap (PG) spectrum was also recently observed for a La-doped Ca1038 compound^10^. In the superconducting state at *T* = 8 and 20 K, the optical conductivity is suddenly suppressed near the frequency of 100 cm^−1^. This is because the opening of the superconducting gap has begun. However, the optical conductivity is not completely suppressed and has a residual Drude component. This phenomenon may be caused by the opening of superconducting gaps with nodes^21,22^. This will be discussed again later.

To analyse the optical conductivity represented by the contribution of the electron bands in the material, we used the standard Drude-Lorentz model as follows:

1 $${\sigma }_{1}(\omega )=\frac{1}{4\pi }[\sum _{j}\frac{{\omega }_{p,j}^{2}}{\frac{1}{{\tau }_{D,j}}\,-\,i\omega }+\sum _{k}{S}_{k}\frac{\omega }{\frac{\omega }{{\tau }_{L,k}}\,+i({\omega }_{0,k}^{2}-{\omega }^{2})}]$$

where ω_*p,j*_ and 1/*τ*_*D,j*_ are the plasma frequency and scattering rate, respectively, for the *j*th free-carrier Drude band. *S*_*k*_, ω_o,*k*_ and 1/*τ*_*L,k*_ are the oscillator strength, resonance frequency and scattering rate, respectively, of the *k*th Lorentz oscillator. As seen from the ARPES results^23,24^, the Ca1048 compound shows a multiband structure similar to those of other iron-based superconductors^10,25^, indicating that at least two Drude components associated with intraband transitions are needed in the optical conductivity fitting. Thus, we used two Drude components here. The optical conductivity data due to high frequency interband transitions are described with six Lorentz components, which are similar to the results of the La-doped Ca1038 compound^20^. The interband transitions are very difficult to map directly to band calculations because bands near the Fermi energy are very complex due to the various hybridizations of Fe-d, As-p and Pt-d bands^26,27^. The parameters for the Drude-Lorentz fitting are summarized in Table 1. The four Lorentz oscillators in the high frequency range are almost independent of temperature, but the two Lorentz oscillators at low frequencies show a weak temperature dependence; as the temperature decreases, the width of the peak decreases slightly and the position of the peak shifts slightly to the high frequency region.

**Table 1:** Parameters obtained by fitting the optical conductivity at 38 and 300 K to the Drude-Lorentz model.

	ω_*P,j*_(cm^−1^)			1/τ_*D,j*_ (cm^−1^)		
	300 K		38 K	300 K		38 K
**Drude spectra parameters**						
Drude 1	2729		2729	68		41
Drude 2	9400		6900	550		175
**Lorentz oscillator parameters**						
	*P*_*k*_ (cm^−1^)		*ω*_0*,κ*_ (cm^−1^)		1/*τ*_*L,k*_ (cm^−1^)	
	300 K	38 K	300 K	38 K	300 K	38 K
Lorentz 1	100	3056	110	144	577	210
Lorentz 2	3678	1658	281	290	315	130
Lorentz 3	5182	5788	774	774	1029	1029
Lorentz 4	11831	10717	1814	1814	2800	2800
Lorentz 5	26211	26265	5043	5043	7000	7000
Lorentz 6	36884	39229	11629	11629	16165	16165

In the Drude spectra parameters, ω_*p,j*_ and 1/*τ*_*D,j*_ are the plasma frequency and scattering rate, respectively. In the Lorentz oscillator parameters, ${P}_{k}=\sqrt{{S}_{k}{Z}_{0}/2\pi }$, where *S*_*k*_ is the oscillator strength and Z_0_ is the impedance of free space, and ω_*o,k*_ and 1/*τ*_*L,k*_ are the resonance frequency and scattering rate of the *k*-th Lorentz oscillator, respectively.

**Figure 2:**
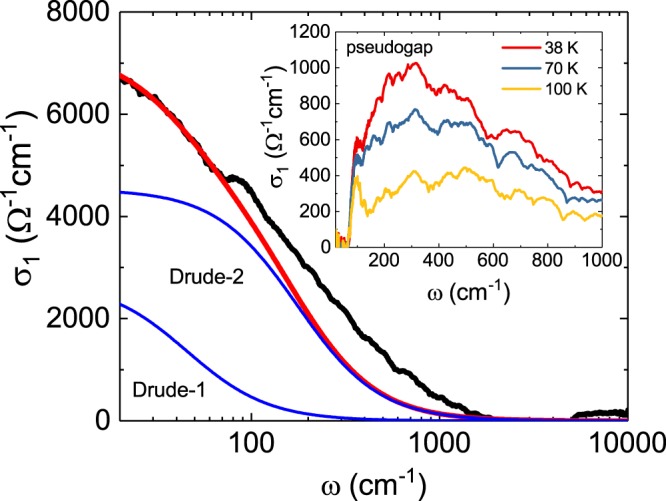
The solid black line shows the optical conductivity with the subtraction of the sum of the Lorentz oscillators determined at *T* = 150 K from the optical conductivity at *T* = 38 K. The blue solid lines represent the responses of Drude-1 and 2 bands, and the red solid line represents the sum of two Drude responses. The inset graph shows the temperature dependence of the pseudogap spectrum obtained by subtracting the red solid line from the black solid line.

The sum of the Lorentz components at 150 K, the temperature at which the pseudogap is not observed, is shown by the red dotted line in Fig. 1(b). To analyse only the PG spectrum in the optical conductivity data, we subtracted the high-frequency Lorentz sum obtained by the previous Drude-Lorentz model fitting from the data at *T* = 38, 70 and 100 K, where a PG was observed. The optical conductivity spectra due to a PG are shown as the solid black lines in Fig. 2. The spectra seem to be the sum of the spectrum of the Drude component and the spectrum of the gap type. To examine this in detail, the optical conductivities below 70 cm^−1^ at 38, 70 and 100 K were fitted using two Drude functions, as shown in Fig. 2. The Drude sums (red solid line) agree well with the low-frequency experimental results. By removing these Drude sums from the PG spectra, we obtained gap spectra, which are plotted in the inset of Fig. 2. It was confirmed that this is a clear gap spectrum. In addition, a temperature dependence for the gap spectrum was clearly observed. The pseudogap spectra show a temperature dependence at which the spectral weight decreases with increasing temperature. However, the change in the absorption edge due to temperature change in the spectra is not observed in the graph. It is not clear whether this is due to an error in the Drude fitting or intrinsic.

**Figure 3:**
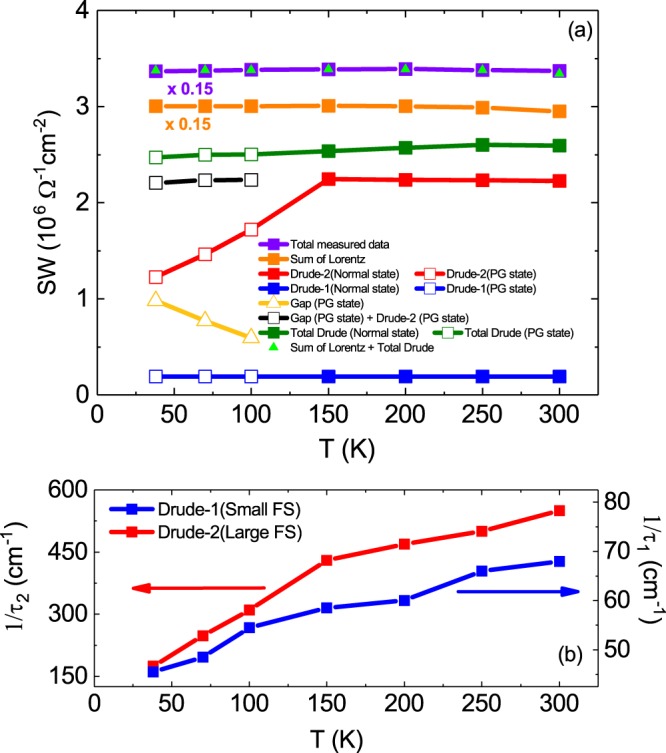
(**a**) The purple solid rectangle and the orange solid rectangle are SW calculated from the measured data and SW for the six Lorentz oscillators obtained with the Drude-Lorentz model, respectively. The blue rectangle and the red rectangle are SWs for the Drude-1 and 2 bands obtained by the Drude-Lorentz model, respectively. The yellow open triangle is SW calculated by PG spectrum. The black open rectangle is the sum of SWs by the Drude-2 band and PG. The green rectangle is the sum of SWs for the Drude-1 and 2 bands and PG. The green triangle is the sum of SWs for six Lorentzian, Drude-1 and 2 bands, and PG. Here, open and solid symbols are for PG and normal states, respectively. (**b**) The graph shows the scattering rates for Drude-1 and 2 bands as a temperature function.

To observe the temperature evolution of the optical conductivity, the spectral weight was calculated as follows:

2 $$SW(T;{\omega }_{c})={\int }_{{0}^{+}}^{{\omega }_{c}}{\sigma }_{1}(\omega ;T)d\omega =\frac{{\pi }^{2}}{{Z}_{0}}{({\omega }_{p})}^{2}$$

where *ω*_*c*_ is the cut-off frequency and Z_o_ and *ω*_*p*_ are the vacuum impedance and plasma frequency, respectively. Figure 3(a) shows the temperature dependence of the spectral weight for each component.

The temperature dependence of the spectral weight (SW) calculated from the experimental optical conductivity (ω < 10000 cm^−1^) is shown in Fig. 3(a) as purple squares. As shown in the figure, SW is constant regardless of temperature and satisfies the sum rule; this means that the sum of SW for the intraband transitions and SW for the interband transitions is constant with respect to the temperature change, since the optical spectra below 10000 cm^−1^ consist of intraband transitions and interband transitions as discussed above. So we calculated SW for each transition using the results of the Drude-Lorentz model fitting. SW for the six Lorentz oscillators plotted in orange squares in Fig. 3(a) was constant regardless of temperature. This means that SW for the Drude responses should also be conserved. As discussed above, there are two Drude bands in intraband transition. SW for the Drude-1 band with a small ω_*p*_ marked by a blue square is constant regardless of temperature. On the other hand, the SW for the Drude-2 band with a large ω_*p*_ marked with a red square is conserved in the normal state, but becomes smaller in the pseudogap state, which is a violation of the sum rule. However, the sum of SW for the Drude-2 band in the pseudogap state and SW (yellow triangles) for the pseudogap structure near 300 cm^−1^ is almost the same as SW calculated in the normal state, which indicates that PG observed in the optical conductivity data is developed in the Drude-2 band and that no spectral weight is transferred to *ω* = 0, unlike in the case of the superconducting Cooper pair. This suggests that the PG feature in this compound follows the preformed Cooper pair model. In the preformed Cooper pair model, there is superconducting (SC) correlation above *T*_*c*_, but due to fluctuation in the SC correlation, the Cooper pairs are destroyed, which leads to the formation of a Drude-free band.

**Figure 4:**
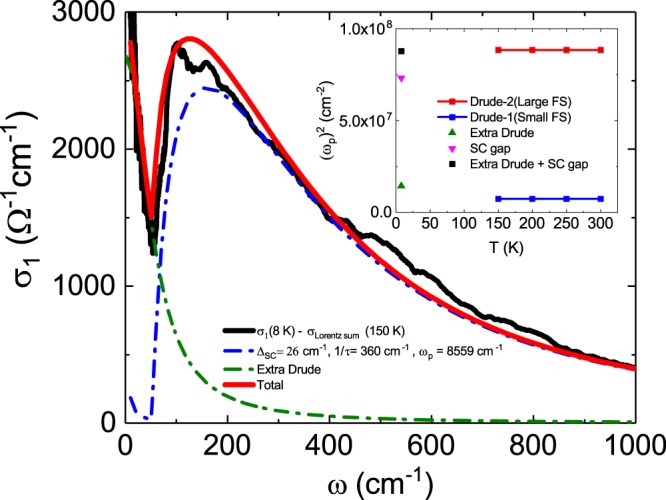
The graph shows the results (the red line) of fitting the *s*-wave Mattis-Bardeen model (the blue dash-dotted line) and Drude model (the green dash-dotted line) to the optical conductivity spectrum (the black line) obtained by subtracting six Lorentz oscillators from the measured optical conductivity in superconducting state, 8 K. The inset shows SWs for the SC gap (down triangle) and Drude response (up triangle) in the superconducting state. The black square is the sum of SWs for the SC gap and the Drude response.

Figure 3(b) shows the temperature dependence of the scattering rate for each Drude band. In both bands, the scattering rate decreases as the temperature decreases, with the Drude-2 band showing a larger scattering rate compared with the Drude-1 band. Figure 4 shows the result of fitting the SC gap optical conductivity spectrum obtained by removing the interband transition spectrum for the superconducting state at *T* = 8 K using the *s*-wave Mattis-Bardeen model^28^. In this fitting, we used one gap (blue dotted line) and an additional Drude function (green dotted line).

In conventional BCS superconductors, optical conductivity would be explained by replacing the normal state Drude term with the Mattis-Bardeen term. However, in the superconducting state, 8 and 20 K, the optical conductivity must add an independent Drude peak in the low frequency range. Thus, the optical conductivity data at 8 and 20 K consist of the same Lorentzians found at 150 K together with the Mattis-Bardeen term and the Drude peak. To get a close look at the data in the low frequency range, we plotted the optical conductivity at 8 K, which removed the six Lorentzians decided at 150 K by the Drude-Lorentz model from the experimental data at 8 K, in Fig. 4. The optical conductivity is well fitted with a Mattis-Bardeen term (the fitting parameters will be discussed later) and an extra Drude peak (ω_*p*_ = 3000 cm^−1^, 1/*τ* = 56 cm^−1^). Note that the measured low-frequency optical conductivity at 8 K are much higher than the optical conductivity due to the quasiparticles thermally excited in the Mattis-Bardeen term which indicates a peak below 50 cm^−1^ drawn in a blue dot-dashed line. This means that the density of the free electrons that produce the low frequency Drude peak is much larger than the density of thermally excited quasiparticles. Quantitatively, the ratio of the density of the free electrons forming the extra Drude calculated from the fitted ω_*p*_ and the density of the quasiparticles formed by the thermal excitation predicted by the BCS theory^1^ to the free electron density in the normal state are calculated to be ~16%, which is similar to the result reported in Ba(Fe_1-x_Co_x_)_2_As_2_^22^, and ~0.4%, respectively. Also note that the measured optical conductivity shows a low frequency upturn with a width of about 70 cm^−1^ which is much wider than the width (~20 cm^−1^) of the low frequency peak due to the thermally created quasiparticles in the Mattis-Bardeen term. Thus, the expression of the Mattis-Bardeen term only fails in the frequency region below the superconducting gap, and a low frequency optical conductivity in the superconducting state requires an additional Drude peak.

The additional Drude peak has also been reported in Co- or K-doped Ba122 compounds^21,22^. Optical spectroscopy studies of these compounds have shown that anisotropy of the SC gap may occur due to Fermi surface reconstruction caused by the spin density wave (SDW), which creates a node in the SC gap to show the residual Drude component. Based on these results, the residual Drude component should not be observed because the SDW is not present in the Ca1048 compound. However, since the residual Drude component is observed in the experimental results, the Ca1048 compound has an SC gap node. It is not possible to exclude correlation, such as short-range order SDW, as a cause for the existence of the SC gap node, as it is difficult to detect. Therefore, further studies are needed to clarify the apparent cause of the SC gap node in Ca1048 compounds.

Optimally La-doped Ca1038 compounds reported previously^10^ were described using two superconducting gaps. The parameters obtained from our fitting results are as follows: an SC gap size of Δ = 26 cm^−1^, a scattering rate of 1/τ = 360 cm^−1^ and a normal-state Drude plasma frequency of *ω*_*p*_ = 8559 cm^−1^. The inset of Fig. 4 shows the temperature dependence of the SC gap and the spectral weights of the two Drude bands in the normal state. The pink inverted triangle and the green triangle represent the spectral weights of the SC gap and the extra Drude band, respectively, and the sum of the two spectral weights is represented by a black square. Since the total weight value is nearly equal to that of the Drude-2 band (red squares), it is clear that both the SC gap and the extra Drude component will occur in the Drude-2 band. The superconducting gap spectra were observed in a frequency region similar to the pseudogap spectra mentioned above. As a result of the similarity of the energy scales for these two gaps and the satisfaction of the sum rule for the spectral weights, it is considered that the pseudogap occurs as a result of a continuous temperature evolution of the SC gap formed in a larger Fermi surface. This continuous change in temperature makes the preformed Cooper pair model scenario clearer.

From the data described above, we conclude that the PG and SC gaps are formed in the Drude-2 band with a large Fermi surface. In this compound, we consider why the SC gap in the Drude-1 band is not observed in IR spectroscopy. As shown in Fig. 3(b), the temperature-dependent scattering rate is much lower in the Drude-1 band than in the Drude-2 band, which suggests that the SC gap in the Drude-1 band is close to the clean limit^29^. According to the s_±_-wave gap theory^30^, a small SC gap is formed in the Drude band with a large Fermi surface, whereas a large SC gap is formed in the Drude band with a small Fermi surface. It is expected that the size of the superconducting gap in our samples will be larger in the Drude band-1 than in the Drude-2 band because this phenomenon is known to be in accordance with the s_±_-wave gap theory for iron-based superconductors. Since the SC gap in the Drude-1 band is close to the clean limit, it is considered that the SC gap in the Drude-1 band will not be observed in optical conductivity measurements. The PG of the Drude-1 band is also not observed because of this clean limit condition.

In recent STM studies on Ca1048 compounds^31^, the SC gap size was observed to be approximately 35 cm^−1^. This result is larger than the size of the SC gap of the Drude-2 band mentioned above, so it is judged to be the SC gap formed from the Drude-1 band. This is because STM can observe the gap regardless of the presence or absence of the clean limit condition for the gap.

In summary, we measured the reflectivity spectra of Ca_10_(Pt_4_As_8_)(Fe_2_As_2_)_5_ single crystals and obtained the optical conductivity spectra. A pseudogap anomaly in these optical conductivity spectra was observed at *T* = 38, 70 and 100 K. The pseudogap anomaly consists of a Drude response and a gap-type absorption. The spectral weights of the absorption spectrum for the gap and Drude bands in the PG gap spectrum were in agreement with those of the Drude-2 band in the normal state and satisfied the sum rule. This means that the Drude-2 band seen in the normal state exhibits a pseudogap in the temperature range below 100 K, which is normal. In the superconducting state at *T* = 8 K, the optical conductivity can be explained by one SC gap by applying the *s*-wave Mattis-Bardeen model^28^, and the increase in the optical conductivity in the low-frequency region caused by the SC gap node is explained by an extra Drude term. We found that the SC gap and PG were formed in the Drude-2 band because the sum rule for the spectral weights of the SC gap, the PG and the extra Drude component was satisfied. Therefore, we conclude that the PG observed above *T*_*c*_ is formed as a result of a continuous temperature evolution of the SC gap from the evidence that the SC gap and PG are formed in the same Drude band and that the two gap spectra are observed in a similar frequency region. These results represent clear experimental evidence that the PG observed in iron-based superconductors is the Cooper pair breaking spectrum as described by the preformed Cooper pair model.

## Methods

A single crystal of Ca_10_(Pt_4_As_8_)(Fe_2_As_2_)_5_ was grown using the Bridgman method with a sealed molybdenum (Mo) crucible and boron nitride (BN). First, the FeAs precursor was synthesized in evacuated quartz ampoules at 1050 °C. Second, the FeAs precursor and the Ca and Pt elements were placed into a BN crucible; then, the BN crucible was placed into a Mo crucible, and a Mo lid was welded onto the crucible using an arc welder in a high-purity Ar-gas atmosphere. Finally, the entire assembly was slowly heated up to 1500 °C in a vacuum furnace consisting of a tungsten meshed heater with a temperature stability of 0.1 °C and kept at this temperature for 72 h; afterwards, the assembly was moved slowly at a rate of 1.8 mm/h in a downward direction for approximately 85 h, and then, finally, slowly cooled down to room temperature. As a result of this process, we obtained high-quality single crystals with a typical size of 2 × 2 × 0.5 mm^3^.

The optical reflectivity spectra *R*(*ω*) of the Ca_10_(Pt_4_As_8_)(Fe_2_As_2_)_5_ single crystals were measured over frequency regions of 70–12000 cm^−1^ and 20–150 cm^−1^ using a Michelson-type and a Martin-Puplett-type rapid-scan Fourier spectrometer. The absolute *R*(*ω*) was determined by an *in situ* Au evaporating method^9^. In this method, the sample position was precisely sought with a feedback method using a He-Ne Laser and a Si-diode detector, reducing the reflectivity error by 0.3%. The real part of the optical conductivity *σ*_1_(*ω*) and the dielectric constant *ε*_1_(*ω*) were derived from the *R*(*ω*) spectra through the K-K transformation. In the K-K transformation, the reflectivity was extrapolated with a Hagen-Rubens function below 20 cm^−1^, with a constant reflectivity from ~1.5 eV (=12000 cm^−1^) to 40 eV, and then with a free-electron approximation *R*(*ω*) ∝ *ω*^−4^.
